# Application Value of Blood Heparin-Binding Protein in the Diagnosis of Acute Exacerbation of Chronic Obstructive Pulmonary Disease

**DOI:** 10.1155/2021/3800211

**Published:** 2021-12-22

**Authors:** Yuan Dong, Xincan Zhou, Ying Zhang, Yan Liu, Xianghui Zhou, Guangming Ren, Qingling Li

**Affiliations:** Affiliated Hospital of China University of Mining and Technology, Xuzhou, Jiangsu 221000, China

## Abstract

**Objective:**

To investigate the expression and clinical significance of serum heparin-binding protein (HBP), C-reactive protein (CRP), and white blood cell count (WBC) in an acute exacerbation of chronic obstructive pulmonary disease (COPD).

**Methods:**

A prospective research model was used to select 63 patients with acute exacerbation of chronic obstructive pulmonary disease who were hospitalized in Xuzhou First People's Hospital from January 2020 to June 2020, and among the chronic obstructive pulmonary disease patients who were followed up in our hospital during the same period, 18 patients were in the stable phase, and 43 healthy patients in our hospital during the same period were selected as the healthy control group. 18 patients with stable chronic obstructive pulmonary disease were selected as the observation group, and 43 healthy people who underwent examination at the same time as the control group. For patients with acute COPD recombination, 5 ml of venous blood was collected according to whether the condition of COPD patients with acute exacerbation was stable or not. 5 ml of venous blood was collected for acute exacerbation. According to their clinical symptoms (such as cough, sputum, and asthma), dyspnea score (MRC score), and pulmonary function (FEV1 and FEV1/FVC), it is determined whether the patient's condition is stable. Patients in the stable COPD group will collect 5 ml of venous blood during the outpatient follow-up, and those in the healthy physical examination group will collect veins on the day of the physical examination. In 5 ml of blood, the levels of HBP and CRP in the blood were measured by the enzyme-linked immunosorbent method and the immunoturbidimetric method, respectively, and the peripheral blood WBC was measured by a blood cell analyzer and its supporting reagents. The differences of the three indicators in each group were statistically analyzed. Normally distributed measurement data were compared using *t*-test, homogeneity of variance of nonnormally distributed measurement data were compared using one-way analysis of variance, uneven variance of nonnormally distributed measurement data were compared using a rank-sum test, and Pearson linear analysis was used for correlation test. Subject working characteristic curve (ROC) was drawn, *P* < 0.05 means the difference is statistically significant, the receiver working characteristic curve was established, and the area under the curve (AUC) was calculated to analyze blood HBP. The value of blood CRP and peripheral blood WBC counts alone or in combination in the diagnosis of acute exacerbations of chronic obstructive pulmonary disease.

**Results:**

The level of blood heparin-binding protein in the acute exacerbation phase was significantly higher than that in the stable phase and healthy controls (*P* < 0.05). In the acute exacerbation stage and stable stage group, the blood heparin binding protein, the percentage of leukocytes, neutrophils, and CRP were detected. There is a correlation between (*P* < 0.05) and a correlation with lung function (FEV1) (*P* < 0.05). The predictive value of heparin-binding protein, white blood cells, neutrophil percentage, CRP, etc. for the acute exacerbation of chronic obstructive pulmonary disease, with the area under the heparin-binding protein curve, is the largest, and compared with the stable phase, the comparison of heparin-binding protein, white blood cells, and CRP is statistically significant (*P* < 0.05).

**Conclusion:**

Heparin-binding protein increases in the stable phase and acute exacerbation phase and is related to other inflammatory factors. It is one of the important inflammatory factors in chronic obstructive pulmonary disease. Heparin-binding protein, white blood cells, CRP, etc. have diagnostic and predictive value for acute exacerbation of chronic obstructive pulmonary disease. Heparin-binding protein has the best predictive result, and the combined index test has a better diagnostic predictive value, which is better than single index detection.

## 1. Introduction

Chronic obstructive pulmonary disease (COPD) is a common and frequently occurring disease, which is considered to be the main public health problem with high morbidity, disability, and mortality. It is predicted that by 2030, COPD will be ranked fourth in the global burden of disease [1], and 50%–70% of the patients' funds will be used for acute exacerbations of COPD. At the same time, the mortality of COPD is related to the frequency and severity of acute exacerbations of chronic obstructive pulmonary disease (AECOPD) [2], and its deterioration is the main determinant of the health status of the disease. COPD is essentially a systemic chronic inflammatory disease [3]. COPD symptoms are common in patients with bronchitis, such as cough and expectoration. At the same time, patients can be accompanied by asthma and wheezing. Chronic cough is often the earliest symptom in patients with chronic obstructive pulmonary disease. With the development of the course of disease, there can be lifelong nonhealing. Obvious symptoms of cough in the morning often occur. Patients can have intermittent coughs or expectoration at night. When the airway is severely blocked, patients usually only have symptoms of dyspnea, not cough. When sputum is sputum, it is usually white mucus or serous foam sputum. Occasionally, it can bring blood threads. During an acute attack, the amount of sputum increases, and there may be purulent sputum. In the early stages of the disease, when going upstairs, patients with chronic obstructive pulmonary disease will have shortness of breath and gradually worsen compared with their peers when going up two or three floors. The most common cause of acute exacerbation of the disease is infection, accompanied by a series of clinical symptoms. At present, the diagnosis of acute exacerbation of COPD mainly relies on clinical exclusion, lacking objective serological indicators, and mainly relies on the symptoms of patients. The therapeutic drugs for chronic obstructive pulmonary disease mainly include bronchodilators, glucocorticoids, immunomodulators, expectorants, and other symptomatic supporting drugs. For the treatment of stable chronic obstructive pulmonary disease, the most commonly used drugs are bronchodilators. Therefore, for COPD, early diagnosis and early intervention of acute exacerbations of COPD are particularly important. In recent years, a variety of inflammatory markers related to diseases have emerged, both in timeliness and diversity. HBP is an inactivated member of the serine protease family [4]. HBP is stored in secretory and eosinophilic granules of neutrophils and released after the activation of neutrophils [5]. In the initial study on its antimicrobial activity, HBP was considered to be an important multifunctional inflammatory medium. Clinical studies have shown that the release of HBP in many bacterial diseases is considered an important marker of severe infection, especially sepsis [6, 7]. Neutrophils play a major role in a variety of cells involved in airway inflammation in COPD, and blood heparin-binding protein is a protein with bactericidal and chemotactic effects released when neutrophils are stimulated [8, 9]. However, the current research on the relationship between blood HBP and COPD is very rare in China and abroad.

## 2. Materials and Methods

### 2.1. Common Data

A prospective study model was used to select 63 patients with acute exacerbation of chronic obstructive pulmonary disease who were hospitalized in Xuzhou First People's Hospital from January 2020 to June 2020, 18 patients with stable chronic obstructive pulmonary disease who were followed up in our hospital during the same period, and 43 healthy people who were examined in our hospital during the same period were selected as the healthy control group. (Table 1) Inclusion criteria are as follows: (1) age >40 years old; (2) in line with the diagnostic criteria of AECOPD and COPD formulated by the GOLD guidelines of the Global Initiative for Chronic Obstructive Pulmonary Disease in 2021, at least two respiratory physicians in our hospital participated in the diagnosis; (3) no hormone or antibiotic drugs were used within 1 month before admission; and (4) other chronic pulmonary diseases were excluded.

### 2.2. Method

The personal information, current medical history, past history, and past drug treatment intervention programs of the subjects were collected. Hematology (white blood cells, neutrophils, CRP, blood HBP, etc.), pulmonary function index (FEV1% pred and FEV1/FVC), symptom score (mMRC and CAT scores), and other related auxiliary examinations were collected.

### 2.3. Specimens Collection and Storage

Fasting venous blood (5 mL) of patients on the next day at stable stage and also of healthy subjects was collected and centrifuged at 3000 rpm for 10 min in the blood vessels collected with sodium citrate anticoagulation. The separated plasma was stored in a refrigerator at −80°C to detect HBP. At the same time, routine blood tests and CRP were performed.

### 2.4. Detect Method

BP detection method, serum HBP using enzyme-linked immunosorbent assay (ELISA). The AZU/HBP ELISA kit was provided by Shanghai Jianglai Biotechnology Co., Ltd. The concentration of the standard substance was in the abscissa, and the OD value was in the ordinate. The standard curve was plotted on the semilogarithmic coordinate paper, and the corresponding concentration was determined from the standard curve according to the OD value of the sample. Specimen testing procedures are strictly in accordance with the kit instructions.

### 2.5. Statistical Analysis

SPSS 23.0 statistical software was used to analyze the differences of three indicators in each group. Normal distribution measurement data were compared using *t* test, homogeneity of variance of nonnormal distribution measurement data were compared using one-way ANOVA, heterogeneity of variance of nonnormal distribution measurement data were compared using a rank-sum test. Subject working characteristic curve (ROC) was drawn, and *P* < 0.05 indicated that the difference was statistically significant. The ROC was established, and the area under the curve was calculated to analyze the value of blood HBP, blood CRP, and peripheral blood WBC count alone and in combination in the diagnosis of acute exacerbation of chronic obstructive pulmonary disease.

## 3. Result

### 3.1. General Data Analysis

This study included 63 cases of acute COPD with recombination, 18 cases of the stable COPD group, and 43 cases of the healthy control group. Their age, gender, height, weight, and pulmonary function were recorded. A few patients were unable to complete the pulmonary function examination due to their severe condition (but previous medical records showed that pulmonary function could help diagnose COPD), and the data were statistically significant (*P* < 0.05). The general data are shown in Table 2.

### 3.2. Comparison of HBP, WBC, Neutrophil, and CRP between AECOPD and Stable Stage

Compared with the patients in the acute phase and stable phase, HBP, neutrophil, and CRP were significantly different (*P* < 0.05) (Table 3).

### 3.3. The Relationship between HBP and WBC, Neutrophil, CRP, FEV1/Pred, and FEV1/FVC in Patients

The results of the correlation between serum HBP and WBC, NC, CRP, and FEV1/pred in patients with acute exacerbation and stable stage showed that HBP was positively correlated with WBC, NC, and CRP and negatively correlated with FEV1/pred in patients with acute exacerbation and stable stage (Table 4)

### 3.4. Diagnostic Value of Serum HBP, WBC, NC, and CRP in AECOPD

**Figure 1:**
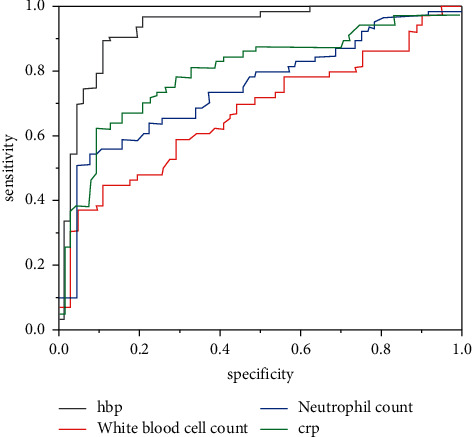
Diagnostic value of serum HBP, WBC, NC, and CRP for AECOPD.

The ROC curve of the AECOPD group was analyzed with the stable COPD group as the control group. As shown in Figure 1, the area under the curve of HBP was 0.927, followed by CRP 0.795, NC 0.749, and WBC 0.688 (Table 5). The probability *P* values of HBP, NC, and CRP were obtained by logistic regression analysis. The stable COPD group was taken as the control group. A ROC curve analysis was performed on the probability *P* values. The area under the curve of the combination of the three indicators was 0.985.

As can be seen from Figure 1, the sensitivity of HBP, CRP, leukocyte count, and neutrophil count gradually increased, the sensitivity and specificity of HBP were higher than those of CRP, leukocyte count, and neutrophil count. Curve data is 1.

## 4. Discussion

Chronic obstructive pulmonary disease is the most common chronic respiratory disease. Acute exacerbation of chronic obstructive pulmonary disease is an important part of chronic obstructive pulmonary disease and one of the key prevention and treatment diseases in the Healthy China 2030 Action Plan. Chronic obstructive pulmonary disease (COPD) is a common, preventable, and treatable chronic airway disease, which is a common disease that seriously endangers human health, seriously affects the quality of life of patients, and is easy to cause death [10]. The latest WHO prediction of mortality and causes of death shows that with the increase of smoking rates in developing countries and the intensification of population aging in high-income countries, the prevalence of the disease will continue to rise in the next 40 years. It is predicted that the number of deaths from the disease and its related diseases will exceed 5.4 million per year by 2060 [10, 11]. There are various factors causing COPD, and its pathogenesis is complex, which has not been clarified. A variety of inflammatory cells are involved in the pathogenesis, including macrophages and neutrophils. Activated inflammatory cells release a variety of inflammatory mediators to act on airway epithelial cells and participate in the induction of an inflammatory response. The neutrophil elastase can cause the destruction of elastin in lung connective tissue, resulting in a series of reactions that eventually cause irreversible lung injury [12]. Respiratory tract infection is an important factor in the pathogenesis and aggravation of COPD, and the common causes of acute exacerbation are viral and/or bacterial infections [13, 14]. At present, many biomarkers, such as white blood cells, neutrophils, CRP, and ESR, are usually detected before the treatment of acute exacerbation, and then the relevant treatment is guided according to the results.

Heparin-binding protein belongs to the trypsin-like serine protease family, which is stored in the phenylenediamine blue particle protein of blood neutrophils and can bind to heparin. Therefore, it is also called as gastrodin [15, 16]. In recent years, a number of studies have shown that this indicator can be used as an early diagnostic marker of infection, and its gradual emergence as a research hotspot is mostly related to its ability of sterilization, chemotaxis, and proinflammatory [17, 18]. Under the stimulation of bacteria, neutrophils secrete and release heparin-binding proteins. Because of its strong bactericidal and chemotactic effects, it can increase the permeability of vascular endothelium, induce vascular leakage, activate monocytes, and lead to a series of inflammatory reactions. Its antimicrobial activity can directly eliminate bacteria [19]. The HBP index of normal healthy people is low, and it is significantly increased in infections, septic shock, sepsis, and other infections, with high sensitivity and specificity [9]. In conclusion, the protein exists in the early stages of inflammation and development and participates in the whole process of disease development. Therefore, it can be used as an early diagnostic marker for infectious diseases. In recent years, many research hotspots have been focused on early infection, especially in sepsis, and the early diagnosis of this protein has great clinical significance. However, there are relatively few studies on acute exacerbations of COPD. In this study, we found that the HBP level was low in the healthy group, increased in the stable COPD group, and significantly increased in the acute reconstitution of COPD. Compared with the traditional indexes such as leukocyte, neutrophil, and CRP, HBP has good advantages in predicting the acute exacerbation of COPD, and the combined prediction of leukocyte, neutrophil, and CRP is an important index for predicting the acute exacerbation of COPD.

C-reactive protein was found in 1930, which is an acute phase reactive protein that can react with pneumococcal C-polysaccharidecoccal bacteria to form a complex. It can bind to the damaged cells, thus stimulating its complement (i.e., the serum protein complex involved in the reaction), and bind to pathogenic microorganisms and their products to cause platelet aggregation and activate lymphocytes and monocytes, thereby promoting the chemotaxis and phagocytosis of neutrophils and macrophages, eventually leading to cell dissolution and rupture, and promoting the absorption of inflammatory cells [20]. When various acute and chronic infections, malignant tumors, trauma, surgical radiation, and other causes of tissue injury occurs the increase of CRP synthesis is related to the acute phase response mediated by cytokines and other media, and is linearly related to the degree of tissue injury. So it becomes an important clinical marker of inflammation and infection. The normal value is  ≤ 8 mg/L. The protein is not affected by radiotherapy and chemotherapy. CRP in both outpatient and inpatient is helpful to reduce the use of antibiotics safely [21]. It has high application value in clinical work. In this study, it was found that in the healthy control group, the stable group and the acute exacerbation group increased in turn, and the difference was statistically significant (*P* < 0.05). In addition, it was also found in the study that the logistics regression analysis was carried out in combination with CRP, HBP, WBC, neutrophils, and other indicators, and the ROC curve analysis was performed on the probability *P* value. It was found that the difference in the combined test of the group of indicators was statistically significant due to the value of a single indicator (*P* < 0.05).

## 5. Conclusion

This study found that, patients with COPD using heparin-binding protein can significantly improve the clinical treatment effect of patients. HBP, CRP, WBC, neutrophils, and other inflammatory indicators can be used as biological indicators of acute exacerbation of chronic obstructive pulmonary disease. Compared with the healthy control group and the stable group, the acute exacerbation group was significantly increased, and the area under the HBP curve was higher than that in the stable group and higher than that in the healthy control group. The difference was statistically significant (*P* < 0.05). The logistic regression analysis of the inflammatory indicators in the group and the ROC curve analysis found that the combined index test was better than the single index test, so it can be considered that the combination of the indicators can be used to judge the acute exacerbation of chronic obstructive pulmonary disease. In the ROC curve, HBP, CRP, sensitivity of leukocyte count, and neutrophil count increased gradually with 1-specificity. The sensitivity of HBP was higher than that of CRP, leukocyte count, and neutrophil count. The curve data was 1. In view of the epidemic factors, the sample size of this study is small. In future studies, it is necessary to further expand the sample size to continue relevant studies.

This study preliminarily confirms that the heparin-binding protein contributes to the early identification of COPD and evaluates the therapeutic efficacy. Further study of the characteristics of heparin-binding protein level in the treatment of COPD is of theoretical significance to guiding the accurate clinical judgment of the condition.

## Figures and Tables

**Table 1:** Observation group, the basic data of the control group.

Group	Example number	Male/female	Age
Observation group	18	12/6	>40
Control group	43	29/14	>40

**Table 2:** Baseline characteristics of general information.

	Healthy controls	Stable group	Acute exacerbation group	Statistic	*P* value
Gender (male : female)	29 : 14	12 : 6	44 : 19	2 = 0.103	0.950
Age	70.767442 ± 13.802755	72.611111 ± 11.319628	73.301587 ± 9.825305	*F* = 0.620803	0.539216
Stature	164.860465 ± 7.379448	164.111111 ± 7.403409	165.587302 ± 7.655137	*F* = 0.308804	0.734901
Body weight	66.279070 ± 11.642135	65.222222 ± 11.471476	61.150794 ± 10.347836	*F* = 3.044480	0.051285

**Table 3:** The Mann–Whitney *U* test of difference between the stable group and the acute exacerbation group.

	Stable group	Acute exacerbation group	Statistic	*P* value
HBP	50.6900 (26.937500, 79.615000)	147.000000 (68.400000, 219.500000)	*Z* = −3.737641	<0.0001
CRP	1.865000 (1.610000, 6.232500)	8.980000 (2.225000, 50.050000)	*Z* = −2.743464	0.006
WBC	6.560000 (5.535000, 7.895000)	6.910000 (5.660000, 9.190000)	*Z* = −1.295110	0.195
NC	4.305000 (3.297500, 5.182500)	5.480000 (3.690000, 6.930000)	*Z* = −2.164172	0.030

**Table 4:** Pearson correlation analysis between WBC, CRP, HBP, NC, and FEV/pred in the stable group and the acute exacerbation group.

		WBC	CRP	HBP	NC	FEV1/pred
WBC	*r* value	1	0.283^*∗*^	0.090	0.947^*∗∗*^	−0.270^*∗*^
	*P* v.		0.011	0.425	0.000	0.015
CRP	r value	0.283^*∗*^	1	0.244^*∗*^	0.327^*∗∗*^	−0.129
	*P* v.	0.011		0.028	0.003	0.250
HBP	*r* value	0.090	0.244^*∗*^	1	0.095	−0.170
	*P* v.	0.425	0.028		0.397	0.129
NC	*r* value	0.947^*∗∗*^	0.327^*∗∗*^	0.095	1	−0.348^*∗∗*^
	*P* v.	0.000	0.003	0.397		0.001
FEV1/pred	*r* value	−0.270^*∗*^	−0.129	−0.170	−0.348^*∗∗*^	1
	*P* v.	0.015	0.250	0.129	0.001	

^*∗*^ *p* < 0.05 and ^*∗∗*^*p* < 0.01.

**Table 5:** ROC curve of acute exacerbation.

Variables	ROC area
HBP	0.927
CRP	0.795
WBC	0.668
NC	0.749

## Data Availability

The data used to support the findings of this study are available from the corresponding author upon request.
